# Haptic Perception in Extreme Obesity: qEEG Study Focused on Predictive Coding and Body Schema

**DOI:** 10.3390/brainsci10120908

**Published:** 2020-11-25

**Authors:** Giuditta Gambino, Giuseppe Giglia, Girolamo Schiera, Danila Di Majo, Maria Stella Epifanio, Sabina La Grutta, Rosa Lo Baido, Giuseppe Ferraro, Pierangelo Sardo

**Affiliations:** 1 Department of Biomedicine, Neuroscience and Advanced Diagnostics (Bi.N.D.), University of Palermo, 90129 Palermo, Italy; giuditta.gambino@unipa.it (G.G.); girolamoschiera@gmail.com (G.S.); danila.dimajo@unipa.it (D.D.M.); rosa.lobaido@unipa.it (R.L.B.); giuseppe.ferraro@unipa.it (G.F.); pierangelo.sardo@unipa.it (P.S.); 2 Euro Mediterranean Institute of Science and Technology-I.E.ME.S.T., 90139 Palermo, Italy; 3 Postgraduate School of Nutrition and Food Science, University of Palermo, 90129 Palermo, Italy; sabina.lagrutta@unipa.it; 4 Department of Psychology, Educational Science and Human Movement, University of Palermo, 90128 Palermo, Italy; mariastella.epifanio@unipa.it

**Keywords:** haptic perception, obesity, EEG, multisensory integration, parietal cortex, temporal cortex, power spectrum analysis, body schema

## Abstract

Haptic perception (HP) is a perceptual modality requiring manual exploration to elaborate the physical characteristics of external stimuli through multisensory integrative cortical pathways. Cortical areas exploit processes of predictive coding that collect sensorial inputs to build and update internal perceptual models. Modifications to the internal representation of the body have been associated with eating disorders. In the light of this, obese subjects were selected as a valid experimental model to explore predictive coding in haptic perception. To this purpose, we performed electroencephalographic (EEG) continuous recordings during a haptic task in normally weighted versus obese subjects. EEG power spectra were analyzed in different time intervals. The quality of haptic performance in the obese group was poorer than in control subjects, though exploration times were similar. Spectral analysis showed a significant decrease in theta, alpha and beta frequencies in the right temporo-parietal areas of obese group, whereas gamma bands significantly increased in the left frontal areas. These results suggest that severe obesity could be characterized by an impairment in haptic performances and an altered activation of multisensory integrative cortical areas. These are involved in functional coding of external stimuli, which could interfere with the ability to process a predicted condition.

## 1. Introduction

Haptic perception (HP) constitutes an integrative process of sensory information mediated by cutaneous and kinesthetic subsystems [1,2] that serves both perceptual and motor goals. Typically, HP implies manual exploration to elaborate the physical characteristics of surfaces and objects such as shape, texture, size, weight and thermal characteristics [3]. Experimental research on HP has focused on the cortical pathways devoted to multisensory integration in the parietal, temporal and occipital lobes [1,2,4,5]. However, compelling evidence points to a larger, widely distributed cortical network with two pathways, respectively devoted to object surface and spatial properties, i.e., a ventral and a dorsal pathway, similar to visual “what and where” streams [6]. Conceptual models and experimental evidence suggest that haptic perception requires a calibration process related to an internal model of the body [7]; moreover, it has been proposed that further sensory signals from external environments are able to adapt this representation. In other words, sensorial inputs are collected in order to build and update internal perceptual models that are later used to predict future sensory states, as postulated by predictive coding theory [8].

Evidence of predictive coding in haptic exploration has been recently provided in a behavioral experiment on contour following [9]. As in other perceptual modalities, generative models are based on precision of sensory inputs. When integrating incongruent sensory signals from two modalities with precise (reliable) and imprecise information, Bayesian integration will favor the high precision modality [10,11]. It has been suggested that imprecise haptic perception can be biased by modifications of the internal model of the body in relation to external stimuli. Indeed, it was found that employing fake hands smaller or larger than the participant’s one can affect the perceived size of haptically explored objects [12]. When the internal model of the hand changed, haptic percept was adjusted accordingly. On this basis, it can be thought that body schema (BS), i.e., dynamic representation of different body parts, plays a role on haptic perception. Therefore, studies on subjects with altered BS, such as patients affected by eating disorders, could be useful in order to evaluate these interactions. 

Previous investigations have reported HP alterations in people affected by eating disorders, namely anorexia nervosa [1,13,14]. Instead, no relevant research has been released about HP in obesity so far. In this context, the use of qEEG allows to evaluate the eventual neural correlates of HP alterations. Indeed, these pathways originate from the transient integration and dynamic links of widely distributed brain districts, thanks to the synchrony over multiple frequency bands, such as theta (4–8 Hz), alpha (8–13 Hz), beta (13–30 Hz) and gamma (≥ 30 Hz) [15]. Cortical oscillatory activity in specific brain areas is a widely used approach for evaluating the activity in cortical regions connected to haptic perception [16,17]. It was evidenced that spectral power density of discrete EEG bands represents a suitable parameter to detect cortical recruitment during the haptic processing of a stimulus object recognition [14], but also to evaluate the implication of predictive coding elaborations. Indeed, experimental data were recently obtained from electrocorticographic recordings in monkeys from cortical areas hierarchically involved in subsequent stages of signal elaboration. These were interpreted exploiting a mathematical model of EEG bands that supported the existence of a Hierarchical Bayesian Network (HBN) across the areas implicated and thus confirming that forward connections were mediated by gamma bands, while the backward from alpha and beta ones [18].

As reports have been published about pathophysiological features found along the underweight-normal weight-overweight “continuum” [19,20,21,22], we pointed to investigate alterations in HP cortical processing in morbidly obese subjects. For this reason, we conducted some pilot experiments that revealed poor haptic performances of extreme obese patients (BMI > 40) carrying out a basic task, which consisted in describing the shape and the constituent material of small objects handled while participants were blindfolded.

Taking all this into consideration, the aim of our current research was to evaluate haptic performances of extreme obese participants in comparison with normal-weight controls. As the investigation of EEG spectral parameters is a suitable method to study stimulus-induced modifications of brain activity [23,24], we collected both behavioral and EEG outcomes, in order to detect electrophysiological correlates of cortical integrative processes and therefore explore the role of predictive coding.

## 2. Materials and Methods

### 2.1. Participants

Twenty-five male right-handed (as measured by Edinburgh Inventory, [25]: LQ > 40) severely obese (BMI > 40 kg/m^2^) patients were consecutively enrolled from the outpatients’ service of obesity and metabolic diseases of the Paolo Giaccone Hospital of the University of Palermo. According to the inclusion criteria enlisted in Table 1, at the starting point of the study, ten obese (OBS) and ten normal-weight control (CTR) participants were included. All subjects showed a normal IQ according to Standard Progressive Matrices (SPM) [26] and similar level of education. None of the enrolled subjects assumed psychoactive drugs nor had family psychiatric history. According to the clinical evaluation by two experienced physicians (a psychiatrist with structured interview based on the DSM-5 [27] criteria and a neurologist), nobody had ongoing psychiatric disorder or showed neurologic symptoms. At the time of assessment, obese subjects had suffered from this condition for at least seven years and were already enlisted for bariatric surgery. The description of the relevant features of enrolled participants is listed in Table 2.

All subjects gave their written consent to the study. The experimental procedure was conducted in accordance with ethical standards of the Declaration of Helsinki and in accordance with the guidelines of Italian Psychological Society (2014) (Ethical approval code: ‘Comitato Etico Palermo1_04.2018’). 

### 2.2. Haptic Task

**Figure 1:**
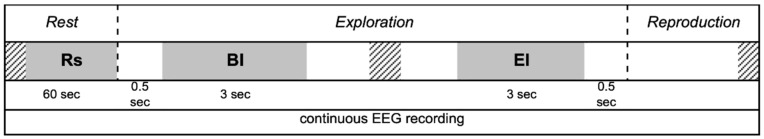
Time course of each single trial, including rest, exploration and reproduction phases during continuous EEG recording. Power spectrum analysis was performed on Rest (Rs), Begin Interval (BI) and End Interval (EI).

According to the protocol by Grunwald [1,13,28], the experimental haptic task was developed in order to induce the activity of cortical areas involved in sensory integration and spatial orienting. In our experiments, participants were asked to explore, with closed eyes and both hands, six different sunken reliefs (13 × 13 cm) presented in random order. A divider screen was interposed between the subject and the item. Participants were allowed to practice for one minute with a sample stimulus prior to the test. At the end of exploration of each test stimulus, they were asked to reproduce, as closely as possible, the explored form by drawing it on a tablet computer (iPad Air 9.7”, vers. 8.4.1, Apple Inc., Cupertino, CA, USA) with the right index finger and open eyes. Therefore, for each item, a single trial included: (1) rest, (2) exploration and (3) reproduction as in Figure 1.

During each trial, participants were comfortably seated on an armchair, with their forearms resting on a wide base in order to freely move their wrists and fingers. Exploration time was not limited and was measured by sensors located under the tables. All sensors were connected to a reliable microcontroller (Arduino uno, https://www.arduino.cc) that, in turn, sent signals to the trigger port of the capturing system in order to guarantee time locked recordings [29].

Participants were not informed about the quality of their reproductions, which were evaluated by a blind experimenter. A scoring scale 1 to 4 was used for the behavioral evaluation: (1) correct reproduction; (2) correct reproduction of the stimulus with one to three mistakes; (3) failure to reproduce the stimulus adequately, correct reproduction of single elements only; (4) failure to reproduce the stimulus or single elements correctly [1,14]. 

### 2.3. EEG Recordings

**Figure 2:**
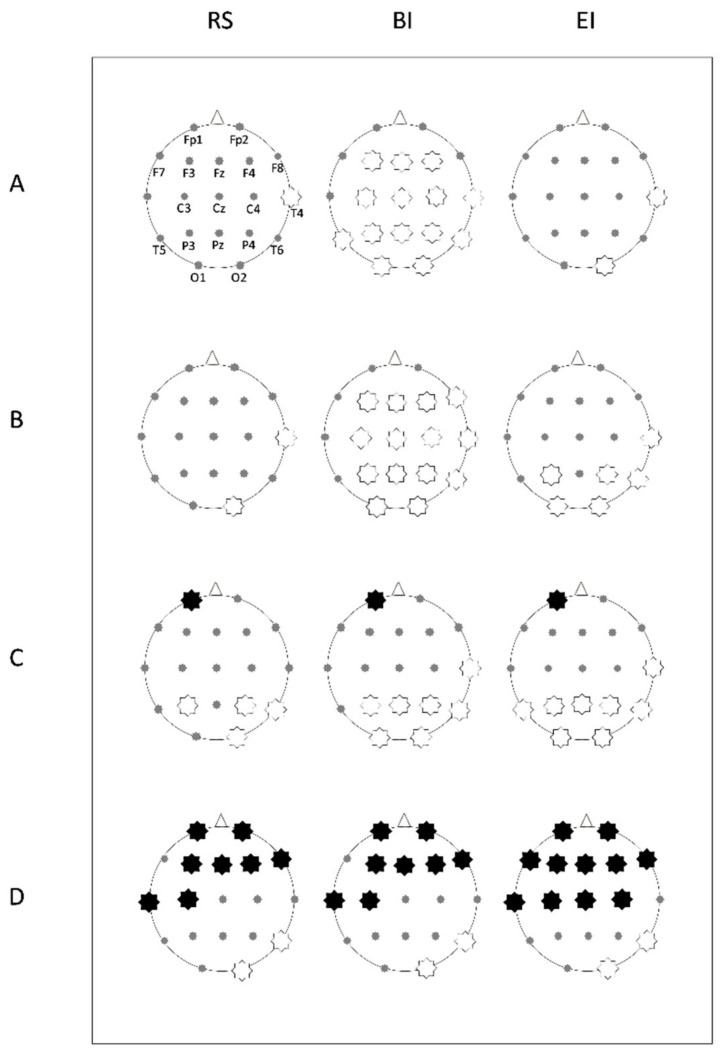
Probability maps for post-hoc power differences between groups in each studied EEG band, during rest (RS), begin interval (BI) and end interval (EI) of exploration. A star corresponding to an electrode of the 10-20 system indicates a statistically significant difference (*P* < 0.01): a white star indicates lower power in the obese (OBS), while a black star indicates higher power in OBS. (**A**) theta, (**B**) alpha, (**C**) beta, (**D**) gamma bands.

A 19-channels digital EEG was continuously recorded during each entire trial (sampling rate = 512 Hz; time constant = 0.3 sec; reference: linked earlobes; ground on the left wrist). Ag-AgCl electrodes were pre-mounted on a cap according with the International 10-20 System [30] on the following positions: Fp1, Fp2, F7, F3, Fz, F4, F8, T3, C3, Cz, C4, T4, T5, P3, Pz, P4, T6, O1, O2 (Figure 2A). Electrode impedance was kept below 15 kOhm. Blinking and eye movements were monitored through bipolar electrooculogram recordings (sampling rate: 512 Hz). All experiments were conducted by the same experimenter in the late morning. EEG data were recorded by a digital EEG apparatus (Esaote Biomedica), stored on a PC-Windows system and processed off-line using the EEGLAB v12 toolbox [31] running on MatLab^®^ R2012 (MathWorks, Natick, MA, USA). Segments containing instrumental and muscular artefacts were manually detected and eliminated from raw EEG. Ocular artefacts were detected by a computerized procedure [32] using Independent Component Analysis [33] and EEG trace was corrected by subtracting the artefactual components without eliminating any further segment. Therefore, only EEG data totally free from artefacts were used for spectral analysis. A digital FFT-based analysis (Welch technique) computed power density of the EEG rhythms with 1 Hz frequency resolution. The following standard band frequencies were studied: theta (4–8 Hz), alpha (8–13 Hz), beta (13–30 Hz) and gamma (30–40 Hz).

The power density of each band was calculated for the following intervals (Figure 1):

Rs Rest (baseline): 60 s with eyes closed before starting exploration.
BI Begin Interval: 3 s of continuous recording extracted after 0.5 s from the beginning of the exploration.
EI End Interval: 3 s of continuous recording ending up 0.5 s before the end of exploration.

### 2.4. Statistical Analysis

Statistical comparisons for age, hand preference, level of education and BMI were performed by t-test for independent groups. Analysis of exploration time (ET) and quality of reproduction (QR) were performed by unpaired, two-tailed t-test between groups. Data are presented as mean ± SD. Before spectral analysis, each power value was log10-transformed to obtain an approximate gaussian distribution. EEG power spectrum was analyzed for each electrode and each stimulus item through a factorial ANOVA in OBS vs. CTR group in Rs, BI and EI for *P* < 0.05 and for statistical power, e.g., *Pr* > 0.5. Data are presented as mean log10-trasformed spectral power (μV2) ± SD.

## 3. Results

### 3.1. Descriptive and Behavioral Data 

**Figure 3:**
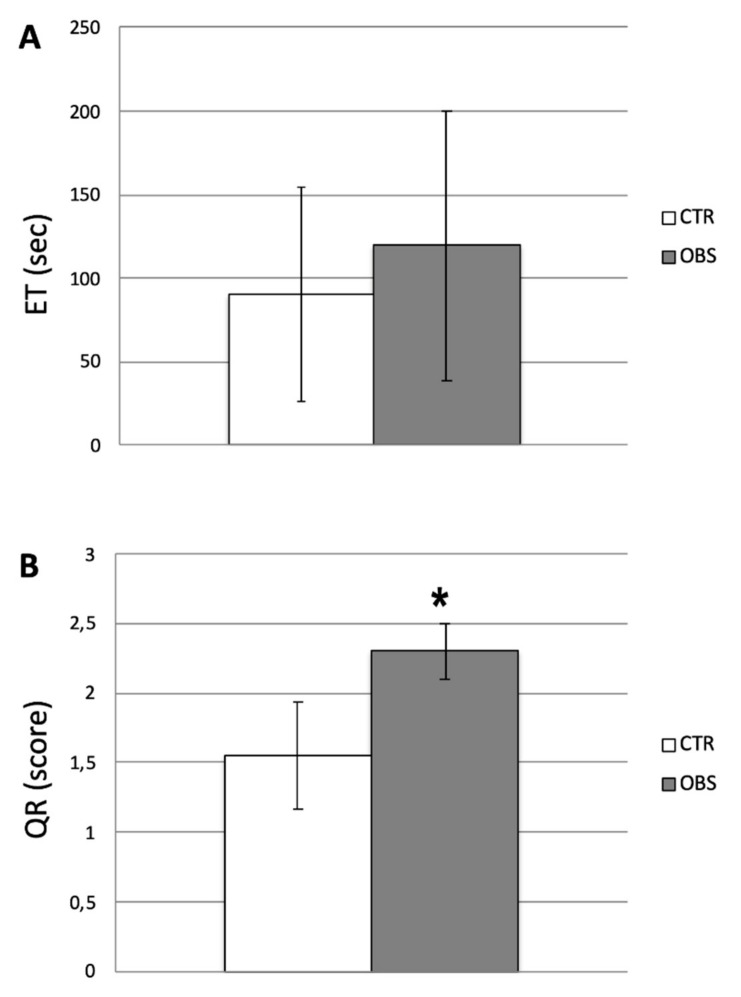
Behavioral evaluations of haptic performances in obese (OBS) vs. control (CTR) groups. (**A**) Mean exploration time (ET) expressed in seconds. (**B**) Mean quality of reproduction (QR) considering the score obtained. Unpaired t-test showed statistical significance for *P* < 0.0001 (*).

**Figure 4:**
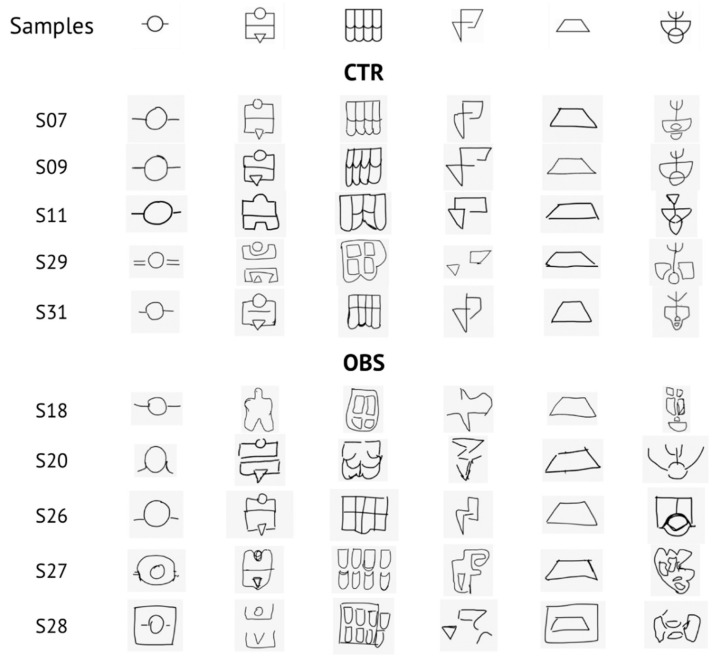
Samples of reproductions in the control (CTR) and obese (OBS) groups (five examples each). In the first line, the six original stimulus items are shown.

Descriptive data of participants’ relevant features are listed in Table 2. As regards behavioral evaluation, ET revealed no statistical differences between groups (CTR: 90.6 sec ± 64.2; OBS: 119.8 sec ± 80.7; Figure 3A), whilst QR score showed a significant increase in OBS versus CTR (CTR: 1.55 ± 0.38; OBS: 2.30 ± 0.20; *t* = 5.513, *df* = 18*, P* < 0.0001; Figure 3B). Samples of reproductions made by OBS and CTR subjects, together with the six original stimulus items, are depicted in Figure 4.

### 3.2. EEG Analysis 

Comparisons of EEG power density were performed for each frequency band for each electrode. This analysis outlined significant differences between groups in the intervals, as reported in probability maps in Figure 2 and summarized as follows.

#### 3.2.1. Theta Power

During Rs, theta power was not significantly different between groups, with the exception of channel T4 (*F*(1,118) = 4.65, *P* = 0.033, *Pr* = 0.562) showing a reduced frequency in OBS versus CTR. 

During BI, the OBS group was characterized by a diffusely lower theta power versus CTR in F3 (*F*(1,118) = 4.84, *P* = 0.029, *Pr* = 0.58), Fz (*F*(1,118) = 4.13, *P* = 0.044, *Pr* = 0.51), F4 (*F*(1,118) = 11.232, *P* = 0.001 *Pr* = 0.93), C3 (*F*(1,118) = 10.48, *P* = 0.0016, *Pr* = 0.91), Cz (*F*(1,118) = 4.15, *P* = 0.043, *Pr* = 0.51), C4 (*F*(1,118) = 12.10, *P* = 0.0007, *Pr* = 0.95), T4 (*F*(1,118) = 16.68, *P* < 0.0001, *Pr* = 0.99), T5 (*F*(1,118) = 6.76, *P* = 0.01, *Pr* = 0.73), P3 (*F*(1,118) = 18.41, *P* < 0.0001, *Pr* = 0.99), Pz (*F*(1,118) = 11.67, *P* = 0.0009, *Pr* = 0.94), P4 (*F*(1,118) = 20.74, *P* < 0.0001, *Pr* = 0.99), T6 (*F*(1,118) = 14.12, *P* = 0.0003, *Pr* = 0.97), O1 (*F*(1,118) = 7.40, *P* = 0.075, *Pr* = 0.78) and O2 (*F*(1,118) = 14.99, *P* = 0.0002, *Pr* = 0.98). 

Such differences were maintained during EI, only in T4 (*F*(1,118) = 8.04, *P* = 0.005, *Pr* = 0.81) and O2 (*F*(1,118) = 5.77, *P* = 0.017, *Pr* = 0.66) (Figure 2A).

#### 3.2.2. Alpha Power

During Rs, alpha power was not significantly different between groups, except for T4 (*F*(1,118) = 5.16, *P* = 0.0248, *Pr* = 0.61) and O2 (*F*(1,118) = 4.87, *P* = 0.029, *Pr* = 0.58) in which alpha was lower in OBS, similarly to what observed for theta power. 

In BI, alpha power was significantly lower in OBS for F3 (*F*(1,118) = 4.38, *P* = 0.038, *Pr* = 0.53), Fz (*F*(1,118) = 7.04, *P* = 0.009, *Pr* = 0.75), F4 (*F*(1,118) = 8.61, *P* = 0.004, *Pr* = 0.84), F8 (*F*(1,118) = 6.07, *P* = 0.015; *Pr* = 0.68), C3 (*F*(1,118) = 9.05, *P* = 0.0032, *Pr* = 0.86), Cz (*F*(1,118) = 7.52, *P* = 0.007, *Pr* = 0.78), C4 (*F*(1,118) = 12.03, *P* = 0.0007, *Pr* = 0.94), T4 (*F*(1,118) = 23.51, *P* < 0.0001, *Pr* = 1), P3 (*F*(1,118) = 15.56, *P* = 0.0001, *Pr* = 0.98), PZ (*F(*1,118) = 9.51, *P* = 0.0025, *Pr* = 0.88), P4 (*F*(1,118) = 15.41, *P* = 0.0001, *Pr* = 0.98), T6 (*F*(1,118) = 10.81, *P* = 0.0013, *Pr* = 0.92), O1 (*F*(1,118) = 7.49, *P* = 0.0071, *Pr* = 0.78) and O2 (*F*(1,118) = 17.92, *P* < 0.0001, *Pr* = 0.99).

Furthermore, during EI the significantly lower alpha power was maintained in channels T4 (*F*(1,118) = 9.009, *P* = 0.0033, *Pr* = 0.86), P3 (*F*(1,118) = 5.56, *P* = 0.02, *Pr* = 0.64), P4 (*F*(1,118) = 7.12, *P* = 0.0087, *Pr* = 0.76), T6 (*F*(1,118) = 5.08, *P* = 0.0259, *Pr* = 0.60), O1 (*F*(1,118) = 4.49, *P* = 0.036, *Pr* = 0.54), O2 (*F*(1,118) = 13.66, *P* = 0.0003, *Pr* = 0.97) (Figure 2B).

#### 3.2.3. Beta Power

Regarding beta band, during Rs the OBS group showed a significantly higher power in Fp1 (*F*(1,118) = 5.8, *P* = 0.017, *Pr* = 0.66), whereas lower power was found in P3 (*F*(1,118) = 4.27, *P* = 0.0409, *Pr* = 0.52), P4 (*F*(1,118) = 10.43, *P* = 0.0016, *Pr* = 0.91), T6 (*F*(1,118) = 14.93, *P* = 0.0002, *Pr* = 0.98), O2 (*F*(1,118) = 15.54, *P* = 0.0001, *Pr* = 0.98). 

Such characteristics remained quite similar during the entire period of exploration. In BI, beta power was increased in OBS in Fp1 channel (*F*(1,118) = 4.31, *P* = 0.039, *Pr* = 0.52); while it was reduced in T4 (*F*(1,118) = 6.14, *P* = 0.014, *Pr* = 0.69), P3 (*F*(1,118) = 8.13, *P* = 0.0051, *Pr* = 0.82), Pz (*F*(1,118) = 8.72, *P* = 0.0038, *Pr* = 0.85), P4 (*F*(1,118) = 20.42, *P* < 0.0001,*Pr* = 0.99), T6 (*F*(1,118) = 21.43, *P* < 0.0001, *Pr* = 0.99), O1 (*F*(1,118) = 10.17, *P* = 0.0018, *Pr* = 0.90), O2 (*F*(1,118) = 21.05, *P* < 0.0001, *Pr* = 0.99). 

During EI, the increase in beta power was maintained in Fp1 (*F*(1,118) = 5.92, *P* = 0.016, *Pr* = 0.67), as well as the reduction in T4 (*F*(1,118) = 5.82, *P* = 0.017, *Pr* = 0.66), T5 (*F*(1,118) = 5.17, *P* = 0.024, *Pr* = 0.61), P3 (*F*(1,118) = 6.41, *P* = 0.012, *Pr* = 0.71), Pz (*F*(1,118) = 10.39, *P* = 0.0016, *Pr* = 0.91), P4 (*F*(1,118) = 25.99, *P* < 0.0001, *Pr* = 1), T6 (*F*(1,118) = 19.14, *P* < 0.0001, *Pr* = 0.99), O1 (F(1,118) = 8.84, *P* = 0.0036, *Pr* = 0.85), O2 (*F*(1,118) = 24.48, *P* < 0.0001, *Pr* = 1) for OBS group compared to CTR (Figure 2C).

#### 3.2.4. Gamma Power

Lastly, OBS showed higher gamma power during Rs in channels Fp1 (*F*(1,118) = 16.92, *P* < 0.0001, *Pr* = 0.99), Fp2 (*F*(1,118) = 14.09, *P* = 0.0003, *Pr* = 0.97), F3 (*F*(1,118) = 9.74, *P* = 0.0023, *Pr* = 0.89), Fz (*F*(1,118) = 6.66, *P* = 0.011, *Pr* = 0.73), F4 (*F*(1,118) = 14.37, *P* = 0.0002, *Pr* = 0.97), F8 (*F*(1,118) = 10.46, *P* = 0.0016, *Pr* = 0.91), T3 (*F*(1,118) = 6.83, *P* = 0.01, *Pr* = 0.74), C3 (*F*(1,118) = 4.42, *P* = 0.037, *Pr* = 0.54). Whilst, lower gamma power was observed in OBS group in T6 (*F*(1,118) = 10.53, *P* = 0.0015, *Pr* = 0.91) and O2 (*F*(1,118) = 8.84, *P* = 0.0036, *Pr* = 0.85). 

At the beginning of exploration, gamma band was higher in OBS in Fp1 (*F*(1,118) = 15.76, *P* = 0.0001, *Pr* = 0.98), Fp2 (*F*(1,118) = 14.17, *P* = 0.0003, *Pr* = 0.97), F3 (*F*(1,118) = 17.72, *P* < 0.0001, *Pr* = 0.99), Fz (*F*(1,118) = 5.14, *P* = 0.025, *Pr* = 0.60), F4 (*F*(1,118) = 12.85, *P* = 0.0005, *Pr* = 0.96), F8 (*F*(1,118) = 6.39, *P* = 0.012, *Pr* = 0.71), T3 (*F*(1,118) = 7.95, *P* = 0.0056, *Pr* = 0.81), C3 (*F*(1,118) = 8.56, *P* = 0.0041, *Pr* = 0.84) and lower in T6 (*F*(1,118) = 10.26, *P* = 0.0017, *Pr* = 0.90) and O2 (*F*(1,118) = 9.55*, P* = 0.0025, *Pr* = 0.88). 

At the end of the exploration, OBS gamma power was enhanced in Fp1 (*F*(1,118) = 15.90, *P* = 0.0001, *Pr* = 0.98), Fp2 (*F*(1,118) = 16.18, *P* = 0.0001, *Pr* = 0.98), F7 (*F*(1,118) = 6.17, *P* = 0.014, *Pr* = 0.69), F3 (*F*(1,118) = 31.29, *P* < 0.0001, *Pr* = 1), Fz (*F*(1,118) = 22.60, *P* < 0.0001, *Pr* = 0.99), F4 (*F*(1,118) = 32.01, *P* < 0.0001, *Pr* = 1), F8 (*F*(1,118) = 13.82, *P* = 0.0003, *Pr* = 0.97), T3 (*F*(1,118) = 12.19, *P* = 0.0007, *Pr* = 0.95), C3 (*F*(1,118) = 20.33, *P* < 0.0001, *Pr* = 0.99), Cz (*F*(1,118) = 8.86, *P* = 0.0035, *Pr* = 0.85), C4 (*F*(1,118) = 6.716, *P* = 0.018, *Pr* = 0.73) and similarly reduced in T6 (*F*(1,118) = 9.31, *P* = 0.0028, *Pr* = 0.87) and O2 (*F*(1,118) = 8.79, *P* = 0.0037, *Pr* = 0.85) (Figure 2D).

## 4. Discussion

Over-eating and obesity are tightly linked to neurobiological, behavioral and cognitive factors such as mood status, addictive behavior, impulsivity and reward processing; cognitive control [19,20,33,34,35,36,37,38,39,40]. Exploring the effects of external modifications to body homeostasis caused by nutritional, neurophysiological or pharmacological tools is a widely applied approach that provides evidence to fundamental processes of the nervous system [41,42,43]. In this context, although cognitive performance in obese people is far from being elucidated [44], obesity has been recently considered as a risk factor for cognitive impairments [45]. Indeed, putative detrimental effects of obesity on cognition are associated with modifications in structural brain properties such as grey matter volume, that could emerge from physiological alterations such as insulin resistance, low-grade inflammation and secondary cardiovascular diseases [45,46,47,48,49].

Behavioral, functional and neurophysiological data point to defective body representation in people affected by eating disorders, such as anorexia nervosa, bulimia nervosa and body dysmorphic disorder [48,49]. Body schema plays a critical role on sensorimotor domain [50], to which HP belongs. Involvement of parietal, temporal and occipital cortices in body representation has been confirmed by numerous results on animal models and humans [4,50,51]. In the last decade, several researches have focused on the neural mechanisms underpinning HP [1,2]. In particular, the study of the putative relationship between eating disorders and alterations of HP would deserve more attention. Grunwald and his group reported data on HP and BS in people affected by anorexia nervosa [1,13,14]. These studies, focusing on EEG theta band, revealed a dysfunction on the right parietal cortex, a brain area crucially involved in cognitive and attentive performances [52]. The dysfunction previously evidenced by Grunwald affected somatosensory integrative processes in patients with a meaningfully altered BS.

To investigate cortical elaboration of haptic stimuli in extreme obesity, we took into account both behavioral and electrophysiological data. In our behavioral evaluation, OBS participants showed a poorer quality of reproduction than normal-weight controls, though exploration times were similar as well as age and education. Since clinical assessment excluded any relevant sensory or motor deficit, the observed impairment in reproduction could be related to altered acquisition or processing of haptic information that hypothetically could influence the consequent sensorimotor transformation. These multisensory integration processes are mainly carried out in the right temporo-parietal cortex and therefore reproduction abilities could be affected by functional modifications in these regions [50,51].

The implication of multi-sensory integration processes in eating disorders is supported by previous studies on anorectic patients. Indeed, distorted proprioception and integration of higher-level visual information can weaken multisensory construction of body schema in the right parietal lobule, similarly to what emerged from studies in anorectic patients [53,54]. Moreover, as previously pointed, haptic perception requires integrative process on both surface properties and spatial information of the explored object, similarly to what and where streams through tactile senses [6]; on this point Lozano-Serra et al. [55] evidenced that anorectic patients performing Ray’s complex figure did not properly integrated complex visual stimuli and showed difficulties in processing and recalling visuo-spatial memory information.

It should be taken into account that although most of the literature focuses on obese children, the study by Waldstein et al. [56] reveals that overweight adults show a slightly significant reduction in performance on the Grooved Pegboard task, thus suggesting a defective dexterity in such people. Haptic perception, in line with the theory of predictive coding, can be seen under the metaphor of “hypothesis testing”. Indeed, the exploration (that is a motor act) is driven by expectations and actively samples the areas that are expected to be salient. In this sense, every element of the sensory-motor circuit constitutes a potential breaking point, including digital dexterity. However, all patients had undergone a standard neurological examination (which includes the finger-tapping task), and none had shown pathological performance allowing gross alterations to be ruled out.

It is worth noting that haptic guidance of exploratory movement has been considered as the counterpart of visual guidance of eye movement, thus leading to the idea that this perceptual modality is strongly based on predictive coding [9]. This hypothesis has been recently confirmed by recording kinematic data collected from finger movement of healthy subjects manually exploring virtual surfaces [9].

In order to explore whether our results can be explained by differences in cortical functioning, we further compared EEG rhythms in experimental groups at rest and during haptic exploration.

In fact, oscillations at different frequencies are essential for connecting brain areas in order to integrate information [15]. In detail, theta oscillations coordinate brain activity, integrate information and underlie working memory [57]; alpha oscillations are considered as a general mechanism of coherent network functioning, specifically gating task-relevant activity in perceptual and attentive tasks [58]; beta band is classically deemed to be involved in the inhibitory control of motor system above all, though recent evidence supports that long-distance beta synchronization is associated with selective attention, perception, learning and sensorimotor integration; lastly, gamma band sustains the parallel processing of complex stimuli and attention [15].

As a first outcome, our data show that the power spectrum in the OBS group is significantly different with respect to healthy controls in right temporo-parietal areas, which are cortical centers connected with several sensory districts, association cortices and subcortical structures [23]. Data showed that, at rest, no differences between groups emerged for both theta and alpha power, indicating a similar basal condition; however, this did not occur for beta and gamma power, revealing significant differences that were maintained in all intervals. Our results showed no differences between groups in the frontal theta power, suggesting that frontal districts in the OBS are still able to elaborate information, though temporo-parietal areas might not sufficiently process relevant sensory input.

**Figure 5:**
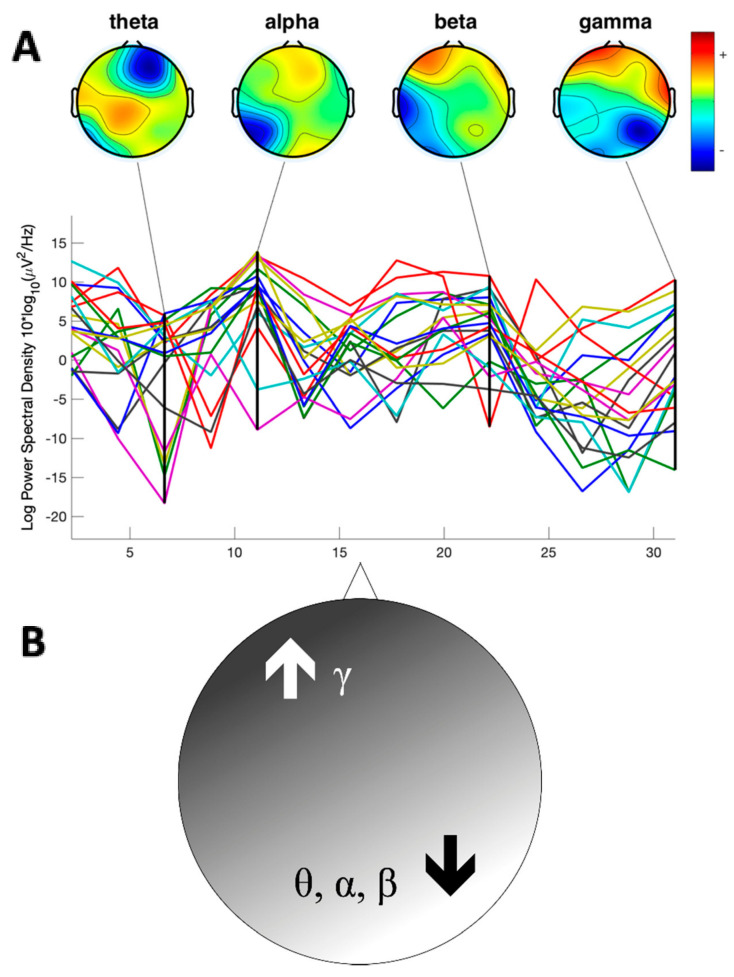
(**A**)**.** Power Spectrum Analysis of a representative obese subject during exploration. Each trace represents power spectrum from each channel (**B**)**.** Schematic representation of the overall differences in the spectral power. In the obese group, a significant and diffuse reduction of the theta (θ), alpha (α) and beta (β) power was individuated in the right posterior areas with respect to controls. In contrast, higher gamma (γ) power was reported in the left anterior areas.

Noteworthy, a further finding is that, at the beginning of exploration, OBS showed a significantly lower theta, alpha and beta power in the right hemisphere in comparison with CTR, especially in temporo-parietal regions. In agreement with the results obtained in previous studies on anorectic patients [13,14], our study revealed that extreme obese participants have lower theta power in the right posterior hemisphere, especially during the beginning of the exploration. Whereas, in contrast to what occurred in the lower frequency bands, gamma activity in the OBS group was enhanced across the left fronto-central channels, in all the experimental intervals (Figure 5A,B).

A possible interpretation of our data could involve predictive coding and related perceptual cortical networks. It is worth noting that haptic guidance of exploratory movement has been considered as the counterpart of visual guidance of eye movement, thus leading to the idea that this perceptual modality is strongly based on predictive coding [9]. This hypothesis has been recently confirmed by recording kinematic data collected from fingers movement of healthy subjects manually exploring virtual surfaces [9].

EEG visualization of cortical oscillations of discrete bands and their propagation has also been considered a hallmark of predictive coding model, i.e., gamma-band seems to be involved in feedforward signal transmission, according to a predictive coding model, the “canonical cortical microcircuit” model [18]. Recently, Van Pelt et al. [59] provided qEEG data describing a cortical network involved in causal inference during an action–perception task comprising prefrontal, temporal and parietal cortices, which has been associated with inference of potential higher-level causes of external events [60]. According to this, it was proposed that top-down predictive signals flow from prefrontal cognitive areas to motion–perception areas, while bottom-up prediction error signals are transmitted in the opposite direction. Downward predictive coding is associated with qEEG correlates represented by beta-band, whereas gamma-band activity is directly related to upward prediction error signals. In detail, beta band increases with the probability of a predicted condition, whilst gamma band increases with prediction error signals. In the light of this, our findings showing that obese patients have low parietal beta and high frontal gamma power, crucially support the idea of an impaired predictive model and an increased error prediction signal in both baseline and during haptic exploration. One could speculate that a defective haptic sensory pathway (low precision input) does not allow a reliable Bayesian internal model, that in turn relies uniquely on visual (higher precision) input, leading to a defective internal representation of the body and to an impaired prediction of incoming input. Given the functional alteration that we found in the right temporo-parietal areas of OBS, we can hypothesize that the difficulty in integration, leading to poor haptic performances, could interfere with body schema formation, similarly to anorectic patients. Nevertheless, considering the limits of the procedure applied, further investigations would be needed to consolidate the speculations proposed in larger series with a higher electrophysiological resolution. Furthermore, individuating eventual modifications in functional connectivity would add insight on the impairment here evidenced in obese subjects.

## 5. Conclusions

The present research runs through the leitmotif of cognitive investigations in the field of disordered eating behaviors. We assessed scarce haptic perception in extreme obesity, associated with noticeable changes in brain rhythms. This study indicates a reduced activation of multi-sensory integration processes, linked to impaired predictability of incoming sensory inputs, and an increase in gamma band-mediated error prediction signals. Our findings could shed light on possible perceptual and cognitive factors linked to altered food intake behavior, in particular, suggesting putative body schema modifications in extremely obese people.

## Figures and Tables

**Table 1:** Inclusion criteria of enrolled subjects, obese (OBS) vs. controls (CTR). LQ (Laterality Quotient). BMI (Body Mass Index).

Inclusion Criteria (Male, Right-Handed (LQ > 40; Age 18–60))	
CTR	OBS
BMI: 18-25 kg/m^2^	BMI > 40 kg/m^2^
healthy	History of obesity for at least 7 years
	No comorbidities
	Scheduled for bariatric surgery at the General Gastroenterologic Surgery- University Hospital “Paolo Giaccone”.

**Table 2:** Descriptive data of participants’ relevant characteristics. Statistical analyses were performed by unpaired t-test in obese (OBS) vs. control (CTR) group. SPM = Standard Progressive Matrices. (*) for *P* < 0.05. All data are presented as mean ± SD.

	CTR	OBS
N	10	10
Age	43.3 ± 5.7	44.4 ± 9.8
BMI *	23.3 ± 3.2	47.6 ± 7.8
Education	13.4 ± 4.3	12.2 ± 3.3
SPM	46.1 ± 7.4	46.7 ± 6.8
